# Extracellular vesicles secreted by cumulus cells contain microRNAs that are potential regulatory factors of mouse oocyte developmental competence

**DOI:** 10.1093/molehr/gaae019

**Published:** 2024-05-14

**Authors:** Giulia Fiorentino, Valeria Merico, Mario Zanoni, Sergio Comincini, Daisy Sproviero, Maria Garofalo, Stella Gagliardi, Cristina Cereda, Chih-Jen Lin, Federica Innocenti, Marilena Taggi, Alberto Vaiarelli, Filippo Maria Ubaldi, Laura Rienzi, Danilo Cimadomo, Silvia Garagna, Maurizio Zuccotti

**Affiliations:** Laboratory of Biology and Biotechnology of Reproduction, Department of Biology and Biotechnology ‘Lazzaro Spallanzani’, University of Pavia, Pavia, Italy; Laboratory of Biology and Biotechnology of Reproduction, Department of Biology and Biotechnology ‘Lazzaro Spallanzani’, University of Pavia, Pavia, Italy; Laboratory of Biology and Biotechnology of Reproduction, Department of Biology and Biotechnology ‘Lazzaro Spallanzani’, University of Pavia, Pavia, Italy; Functional Genomics Laboratory, Department of Biology and Biotechnology ‘Lazzaro Spallanzani’, University of Pavia, Pavia, Italy; IFOM, IFOM—The FIRC Institute of Molecular Oncology, Milan, Italy; Molecular Biology and Transcriptomics Unit, IRCCS Mondino Foundation, Pavia, Italy; Molecular Biology and Transcriptomics Unit, IRCCS Mondino Foundation, Pavia, Italy; Department of Pediatrics, Center of Functional Genomics and Rare Diseases, Buzzi Children’s Hospital, Milan, Italy; Centre for Reproductive Health, Institute for Regeneration and Repair, University of Edinburgh, Edinburgh, UK; IVIRMA Global Research Alliance, GENERA, Clinica Valle Giulia, Rome, Italy; IVIRMA Global Research Alliance, GENERA, Clinica Valle Giulia, Rome, Italy; IVIRMA Global Research Alliance, GENERA, Clinica Valle Giulia, Rome, Italy; IVIRMA Global Research Alliance, GENERA, Clinica Valle Giulia, Rome, Italy; IVIRMA Global Research Alliance, GENERA, Clinica Valle Giulia, Rome, Italy; Department of Biomolecular Sciences, University of Urbino “Carlo Bo”, Urbino, Italy; IVIRMA Global Research Alliance, GENERA, Clinica Valle Giulia, Rome, Italy; Laboratory of Biology and Biotechnology of Reproduction, Department of Biology and Biotechnology ‘Lazzaro Spallanzani’, University of Pavia, Pavia, Italy; Laboratory of Biology and Biotechnology of Reproduction, Department of Biology and Biotechnology ‘Lazzaro Spallanzani’, University of Pavia, Pavia, Italy

**Keywords:** miRNA, extracellular vesicles, cumulus cells, cumulus–oocyte communication, oocyte developmental competence, mouse

## Abstract

The role of cumulus cells (CCs) in the acquisition of oocyte developmental competence is not yet fully understood. In a previous study, we matured cumulus-denuded fully-grown mouse oocytes to metaphase II (MII) on a feeder layer of CCs (FL-CCs) isolated from developmentally competent (FL-SN-CCs) or incompetent (FL-NSN-CCs) SN (surrounded nucleolus) or NSN (not surrounding nucleolus) oocytes, respectively. We observed that oocytes cultured on the former could develop into blastocysts, while those matured on the latter arrested at the 2-cell stage. To investigate the CC factors contributing to oocyte developmental competence, here we focused on the CCs' release into the medium of extracellular vesicles (EVs) and on their miRNA content. We found that, during the 15-h transition to MII, both FL-SN-CCs and FL-NSN-CCs release EVs that can be detected, by confocal microscopy, inside the zona pellucida (ZP) or the ooplasm. The majority of EVs are <200 nm in size, which is compatible with their ability to cross the ZP. Next-generation sequencing of the miRNome of FL-SN-CC versus FL-NSN-CC EVs highlighted 74 differentially expressed miRNAs, with 43 up- and 31 down-regulated. Although most of these miRNAs do not have known roles in the ovary, *in silico* functional analysis showed that seven of these miRNAs regulate 71 target genes with specific roles in meiosis resumption (N = 24), follicle growth (N = 23), fertilization (N = 1), and the acquisition of oocyte developmental competence (N = 23). Overall, our results indicate CC EVs as emerging candidates of the CC-to-oocyte communication axis and uncover a group of miRNAs as potential regulatory factors.

## Graphical Abstract

**Graphical Abstract:**
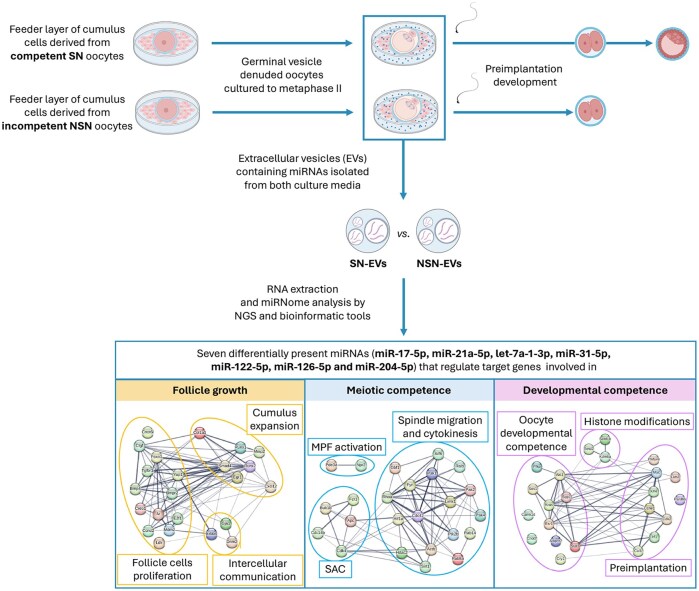
**Identification of seven CC-derived microRNAs and their putative role in the regulation of genes involved in follicle growth, oocyte meiotic resumption, and acquisition of oocyte developmental competence.** EVs: extracellular vesicles; NSN: not surrounding nucleolus; SN, surrounded nucleolus. Created with BioRender.com.

## Introduction

The primary function of the ovary is to produce fertilizable eggs that can support embryonic development. This is accomplished through the process of folliculogenesis, which begins in mice shortly after birth. Groups of primordial oocytes become surrounded by a layer of follicle cells, establishing a permanent connection with germ cells through thin cytoplasmic transzonal projections (Anderson and Albertini, 1976; Baena and Terasaki, 2019; Clarke, 2022). This physical relationship is maintained throughout the folliculogenetic process and is only dissolved at the time of ovulation. Upon puberty, at each reproductive cycle, a group of primary follicles is recruited and begins to grow. However, only a small minority of these follicles reach the fully-grown antral stage and are ovulated, while the majority are eliminated through atresia (Fiorentino *et al.*, 2023).

Follicle growth is regulated by a finely co-ordinated bi-directional communication between the oocyte and the surrounding granulosa and cumulus cells (GCs and CCs, respectively) that results from a paracrine exchange of nutrients and signalling molecules. Over the last 30 years, our understanding of the reciprocal communication within the cumulus–oocyte–complex (COC) has significantly improved (Russell *et al.*, 2016). It is known that the female gamete plays a leading role in co-ordinating the follicle developmental programme through the release of growth factors (Eppig *et al.*, 2002; Gilchrist *et al.*, 2006; Su *et al.*, 2009). Oocytes release factors that regulate processes such as formation of the primordial follicle pool (Soyal *et al.*, 2000), GC metabolism (Buccione *et al.*, 1990; Sugiura *et al.*, 2007) and proliferation (Vanderhyden *et al.*, 1992; Vitt *et al.*, 2000; Otsuka *et al.*, 2000), primary-to-secondary and pre-antral-to-antral transitions (Diaz *et al.*, 2007; Dong *et al.*, 1996; Galloway *et al.*, 2000; Latham *et al.*, 2004; Orisaka *et al.*, 2006), and CC expansion after LH surge (Dragovic *et al.*, 2007; Joyce *et al.*, 2001; Su *et al.*, 2004).

Our understanding of the role played by the somatic component of the COC in the communication axis with the oocyte is still fragmented. CCs produce factors that are involved in the arrest of oocyte transcriptional activity and of its ooplasm and meiotic maturation (Eppig, 1991). However, the role of CCs in the acquisition of oocyte developmental competence is still unclear.

Within the follicle, small and large molecules, ribonucleoproteins, proteins, and organelles are directly transferred among the oocyte, CCs, and GCs (Macaulay *et al.*, 2016; Uzbekova *et al.*, 2020; Fiorentino *et al.*, 2023). They can also be delivered through extracellular vesicles (EVs) (da Silveira *et al.*, 2012; Navakanitworakul *et al.*, 2016; Hung *et al.*, 2015, 2017), which contain several different molecules including lipids, proteins, and nucleic acids. As previously documented, EVs containing microRNAs (miRNAs) or long noncoding RNAs are produced by follicle cells and are abundantly released into the follicular fluid of equine (da Silveira *et al.*, 2012), bovine (Sohel *et al.*, 2013) and human (Diez-Fraile *et al.*, 2014) follicles. There is increasing evidence that miRNAs contribute to the regulation of specific key pathways operating during follicle growth, dominance, or atresia, as well as the acquisition of oocyte developmental competence (Machtinger *et al.*, 2016; Assou *et al.*, 2013; Tesfaye *et al.* 2018; Alexandri *et al.* 2020; Innocenti *et al.*, 2022).

To enhance our comprehension of the contribution of CCs to oocyte developmental competence, we recently established a platform to co-culture mouse oocytes with CCs: CC-denuded germinal vesicle (GV) antral oocytes (DOs) were cultured to the metaphase II (MII) stage on a feeder layer of CCs (FL-CCs) isolated from developmentally competent (surrounded nucleolus, SN; Zuccotti *et al.*, 1998) or incompetent (not surrounding nucleolus, NSN) oocytes (Cavalera *et al.*, 2019). The efficiency of the GV-to-MII transition and the acquisition of developmental competence to blastocyst were higher when DOs were cultured on a FL prepared with CCs derived from developmentally competent SN (FL-SN-CCs + DOs) than from incompetent NSN (FL-NSN-CCs + DOs) oocytes or in the absence of a FL. The study showed that FL-SN-CCs significantly contribute to the acquisition of the oocyte’s meiotic and developmental competence, with a developmental rate to blastocyst equal to that obtained with the maturation of intact COCs. Lack of this support, either in the absence of CCs or in the presence of FL-NSN-CCs, resulted in preimplantation developmental failure with embryos arresting at the 2-cell stage (Cavalera *et al.*, 2019).

In summary, these previous experiments have demonstrated the critical role of CC origin, i.e. whether they were isolated from fully-grown COCs enclosing developmentally competent or incompetent oocytes, in addition to their presence (Chen *et al.*, 2013). This result prompted further investigation into the FL-SN-CCs factors which contribute to the acquisition of oocyte developmental competence.

The present study focuses on FL-CC production and release into the culture medium of EVs containing noncoding miRNAs, as emerging candidates of CC-to-oocyte communication. To this end, FL-SN-CCs + DOs or FL-NSN-CCs + DOs were cultured for 15 h and the following analyses were conducted: the amount of EVs released into the medium and the amount positive for the CD9 surface marker were recorded using imaging flow cytometry; EV morphology and size were characterized by transmission electron microscopy (TEM); the passage of EVs through the zona pellucida (ZP) and their internalization into the oocyte were imaged using confocal microscopy; the miRNome of the released EVs was profiled using next-generation sequencing (NGS); and putative miRNA target genes and pathways involved in the acquisition of oocyte meiotic and developmental competence were identified through bioinformatic analysis.

## Materials and methods

### Animals and reagents

CD1 female mice aged 4 weeks were purchased from Charles River (Lecco, Italy). Animals were maintained under controlled conditions of 22°C, 60% air humidity, and a 12:12 h light/dark cycle. The research on mice was conducted with permission from the Ministry of Health (No. 1100/2016-PR) in accordance with the guiding principles of European (No. 2010/63/UE) and Italian (No. 26/2014) laws protecting animals used for scientific research. Unless otherwise specified, reagents were purchased from Merck (Sigma-Aldrich, St Louis, MI, USA).

### Preparation of FL-SN-CCs and FL-NSN-CCs

The protocol for preparing FL-SN-CCs and FL-NSN-CCs is detailed in Cavalera *et al.* (2019). Briefly, a total of 80 6-week-old females were injected with 10 IU Folligon (Intervet Productions Srl, Aprilia, Italy) 48 h prior to sacrifice. Forty-eight mice were used for imaging flow cytometry and TEM experiments, 16 for confocal microscopy and 16 for NGS. After ovary isolation, the ovarian surface was punctured using a sterile 21G needle in HEPES-buffered MEM–Glutamax medium (Thermo Fisher Scientific, Waltham, MA, USA) supplemented with 15% foetal bovine serum (FBS), 25.3 mg/ml sodium pyruvate, 100 IU/ml penicillin, 75 μg/ml streptomycin, and 1 mg/ml fetuin. Only fully-grown antral COCs with more than three layers of CCs were collected. Following their isolation, individual COCs were washed twice in drops of fresh α-MEM-Glutamax medium supplemented with 15% FBS, 25.3 mg/ml sodium pyruvate, 100 IU/ml penicillin, 75 μg/ml streptomycin, and 1 mg/ml fetuin. After separating CCs from their enclosed oocytes by gently pipetting each single COC in and out through mouth-controlled sterile glass hand-pulled Pasteur micropipettes, oocytes were individually placed into 5 µl droplets of M2 medium containing 0.05 µg/ml Hoechst 33342 and incubated for 15 min at room temperature in the dark. Stained oocytes were observed using an AX70 microscope (Olympus, Shinjuku, Japan) under ultraviolet fluorescent light and, depending on their chromatin organization, classified as SN or NSN. SN oocytes are surrounded by twice as many CCs as NSN oocytes (i.e. 2060 ± 727 vs 1322 ± 416, respectively; *P* < 0.02; Cavalera *et al.*, 2019). Thus, to prepare FLs beginning with a similar number of cells, CCs, derived from 15 SN or 30 NSN oocytes, were cultured in a single well of a 96-well plate (PerkinElmer, Waltham, MA, USA) containing 300 μl α-MEM–Glutamax medium (supplemented with 15% FBS, 25.3 mg/ml sodium pyruvate, 100 IU/ml penicillin, 75 μg/ml streptomycin and 1 mg/ml fetuin) at 37.5°C, 5% CO_2_ in air for 72 h or after at least 70% cell confluence. The medium was replaced every 24 h.

### Production of EVs, their isolation, and characterization

The production of EVs from FL-CCs was done in the following four experimental conditions: feeder layer of CCs derived from SN oocytes (FL-SN-CCs); FL-SN-CCs + DOs; feeder layer of cumulus cells derived from NSN oocytes (FL-NSN-CCs); and FL-NSN-CCs + DOs. Eight to 10 fully-grown unclassified antral DOs were transferred onto each single FL-SN-CCs or FL-NSN-CCs in 300 µl α-MEM–Glutamax medium supplemented with 5% exosome-depleted FBS (Thermo Fisher Scientific), 25.3 µg/ml sodium pyruvate, 100 IU/ml penicillin, 75 µg/ml streptomycin, 50 mU/ml FSH, 5 ng/ml epidermal growth factor, and matured at 37°C and 5% CO_2_ in air for 15 h. EV isolation was performed from three independent experiments after 15 h culture as follows: 300 μl medium covering the FL was transferred into ultra-clear centrifuge tubes and centrifuged at 100 000 × g for 60 minutes at 4°C using an Ultra-High-Speed Centrifuge Optima MAX-XP (Beckman Coulter, Brea, CA, USA). The resulting pellet was resuspended in 50 µl of sterile ice-cold D-PBS or exosome-depleted α-MEM–Glutamax medium for EV or miRNA characterization, respectively (see below).

### Imaging flow cytometry analysis of EVs

EVs from FL-SN-CCs, FL-SN-CCs + DOs, FL-NSN-CCs, FL-NSN-CCs + DOs, or exosome-depleted α-MEM–Glutamax medium-only samples were labelled with a rabbit anti-CD9 antibody (Cell Signaling, #13174, Danvers, MA, USA), fluorescently conjugated with the DyLight488 labeling kit (BioRad, Hercules, CA, USA). To eliminate aggregates, CD9 conjugated DyLight488 antibody (0.1 μg) was centrifuged for 10 min at 17 000 × g and, then, incubated for 1 h at room temperature with EVs and 1% (v/v) bovine serum albumin. Then, CD9 stained particles were purified from unstained dyes using Exosome spin columns (MW 3000; Thermo Fisher Scientific) as previously described (Slivinschi *et al.* 2022).

The EVs were analyzed using the ImageStreamX MarkII flow cytometer (ISX; Amnis/Luminex, Austin, TX, USA) equipped with three lasers (100 mW 488 nm, 150 mW 642 nm, 70 mW 785 nm). To detect fluorescent EVs, a 488 nm laser was set to 10 mW power and data were acquired using a 60× magnification (NA = 0.9; DOF = 2.5 µm; core size = 7 µm). CD9 florescence signals were collected using channel 2 (480–560 nm filter) while channel 6 (745–800 nm filter) was used for scatterplot detection. Standard sheath fluid (D-PBS) without further filtration was used in all measurements. Negative controls consisted of detergent lysis controls, buffer controls without particles, and unstained antibody samples.

### Transmission electron microscopy analysis of EVs

EVs derived from the four experimental conditions were visualized using TEM following the methods described in Comincini *et al.* (2017) and Martinelli *et al.* (2020). Specifically, 20 μl drops of D-PBS, containing the isolated EVs, were placed onto a Parafilm sheet (Sigma-Aldrich), and, for each drop, a 300-mesh nickel grid (covered with a Formvar-carbon film) was allowed to float for 5 min. Then, the grids were blotted rapidly with filter paper and negatively stained with a 2% phosphotungstic acid solution, pH 7.0, for 60 s. After blotting on paper, they were observed directly on a Zeiss EM900 electron microscope (Zeiss, Oberkochen, Germany) operating at 80 kV.

### Confocal microscope analysis

Fifty microliters of EV suspension from FL-SN-CCs + DOs, collected as described above, and 50 µl of exosome-depleted α-MEM–Glutamax medium without EVs were stained with the fluorescent dye PKH67 (Martinelli *et al.*, 2020). The resulting samples, together with control samples of exosome-depleted α-MEM–Glutamax medium without EVs and without PKH67 or exosome-depleted α-MEM–Glutamax supplemented with PKH67-unlabelled EVs, were purified from the excess of unlabelled PKH67 with Exosome Spin Columns MW 3000 (Invitrogen, Waltham, MA, USA). Then, samples were centrifuged at 100 000 × g for 60 min at 4°C to pellet EVs and pellets were resuspended in 300 µl α-MEM–Glutamax medium supplemented with 5% exosome-depleted FBS. At the end of this procedure, the dilution from 50 to 300 µl medium led to a reduction of EV concentration by a factor of 6 (∼91.000 ± 22.000 particles/µl). To evaluate EVs crossing through the ZP, 8–10 DOs were cultured for 15 h in these four media until they reached MII. After IVM, MII oocytes were fixed in 4% paraformaldehyde in PBS for 30 min, rinsed three times in PBS/0.1% Tween-20 for a total of 15 min, and counterstained with 0.2 μg/ml DAPI in PBS for 5 min at room temperature. Finally, oocytes were observed under a SP5 confocal microscope (Leica, Wetzlar, Germany).

### RNA extraction

RNA was extracted from EVs released by FL-SN-CCs + DOs or FL-NSN-CCs + DOs, in triplicate experiments, using the miRNeasy Micro Kit (Qiagen, Hilden, Germany) according to the manufacturer’s instructions. Briefly, after EV isolation (see above), EVs were homogenized in QIAzol Lysis Reagent. Following the addition of chloroform, the homogenate was separated into aqueous and organic phases by centrifugation at 13 000 × g for 15 min at 4 °C. The RNA-containing aqueous phase was extracted using ethanol and the sample transferred to the RNeasy MinElute spin column, where the total RNA binds to the membrane while phenol and other contaminants are washed away. High-quality RNA was then eluted in 14 μl of RNase-free water and quantified using Tape Station (Agilent, Santa Clara, CA, USA). The samples were stored at −80°C.

### Next-generation sequencing and q-RT-PCR

For miRNA sequencing, libraries were prepared using the Small RNA-Seq Library Prep kit (Lexogen, Vienna, Austria). The RNA was initially ligated to a 3′ adaptor, and excess 3′ adaptor was removed by column purification. The same step was repeated for the 5′ end. In the second step, the RNA, flanked by 5′ and 3′ adapters, was converted to cDNA and amplified by PCR. The resulting library was cleaned and concentrated using a protocol based on the use of magnetic beads (Lexogen). This step removed any artefacts that could reduce the amplification power of the libraries and, consequently, the extracted sequences. The quality of each library was then assessed using the 2100 Bioanalyzer and the High sensitivity dsDNA Qubit assay for fluorimetric quantification. NGS technologies (Illumina Genome Analyzer and the NextSeq 500/550 High Output v2.5 kit; 150 cycles; Illumina, San Diego, CA, USA) were used for sequencing. RNA processing was performed with Illumina NextSeq 500 Sequencing.

The miRCURY LNA RT Kit (Qiagen) was used for reverse transcription, following the manufacturer’s instructions. Briefly, 4 µl of total RNA, including miRNA, was incubated at 42°C for 1 h for reverse transcription. The enzyme was then inactivated by incubating for 5 min at 95°C. The resulting cDNA was immediately stored at −20°C. Four out of 20 µl of the resulting cDNA product were amplified in duplicate by quantitative RT-PCR (qPCR) in a 10 μl reaction mixture. The qPCR was performed on cDNA from four independent experiments using the miRCURY LNA SYBR Green PCR Kit (Qiagen) in a Rotorgene 6000 (Corbett Life Science, Mortlake, Australia) thermocycler. The primers used are listed in Supplementary Table S1. After an initial incubation step of 2 min at 95°C to activate the QuantiNova DNA Polymerase, a two-step cycling (denaturation for 10 s at 95°C, followed by combined annealing/extension cycles at 56°C for 60 s) was repeated for 50 cycles. hsa-miR-16-5p was used as an internal control for normalization.

### Diana tools identification of miRNA target genes

To identify the target genes of the miRNAs selected in this study, the ‘mirPath v.3’ server available on the DIANA tools website (https://dianalab.e-ce.uth.gr/html/mirpathv3/index.php?r=mirpath; Vlachos and Hatzigeorgiou, 2017) was used. For each miRNA, the list of target genes was searched by entering *Mus musculus* as the reference species and using all the databases provided by the site: TarBase v7.0, microT-CDS (v5.0), and TargetScan. In particular, TarBase v7.0 is the largest manually curated database based on experimentally demonstrating miRNA–gene interactions. The microT-CDS (v5.0) and TargetScan databases use algorithms to predict the binding affinity between the studied miRNA and target genes.

### KEGG identification of pathways related to miRNA target genes

To relate the list of identified genes to their associated pathways, a bioinformatics analysis was conducted using the STRING software (Search Tool for the Retrieval of Interacting Genes/Proteins; https://string-db.org/) in combination with the KEGG (Kyoto Encyclopedia of Genes and Genomes) database. Genes associated with pathways relevant to the processes of folliculogenesis and oocyte maturation were further investigated through a bibliographic search on PubMed without temporal limits. Specifically, each gene of interest was cross-referenced with the following keywords: ovary (12 759 articles found), folliculogenesis (771), ovarian follicle (5504), CC (1123), oocyte (5960), oogenesis (873), meiosis (221), or preimplantation (1514). References were further selected within the class of Mammals and duplicates were manually eliminated.

### Statistical analysis

Imaging flow cytometry results were processed using IDEAS software (version 6.2; Amnis, Seattle, WA, USA). ANOVA test was performed using RStudio and data were considered statistically significant when *P* < 0.05. NGS fastQ files, generated through Illumina bcl2fastq2 from the raw sequencing reads, were analyzed with the DESeq statistical RStudio package (RStudio, PBC, Boston, MA, USA) to identify differentially expressed miRNAs. The transcripts were selected for further analysis if they met the criteria of log2 ≥ 1 and false discovery rate (FDR) ≤0.1, indicating differential expression. qPCR results were analyzed using the Student’s *t*-test (RStudio) and considered statistically significant when *P* < 0.05.

## Results

### Detection and characterization of EVs released into the medium by FL-CCs

**Figure 1.**
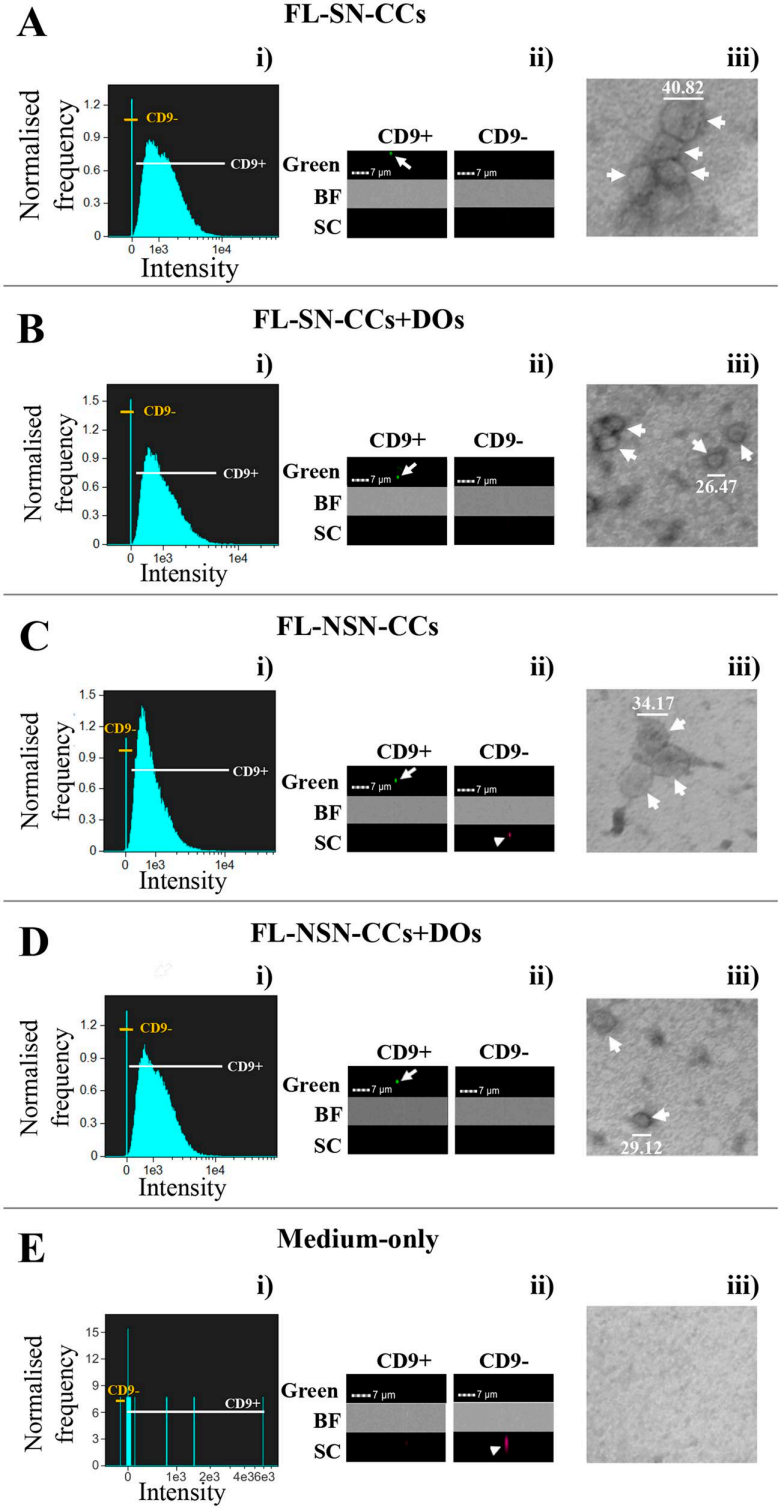
**Imaging flow cytometry and transmission electron microscopy characterization of extracellular vesicles released in the culture medium by feeder layers of mouse cumulus cells isolated from developmentally competent or incompetent oocytes in the presence or absence of denuded oocytes.** (**A**) FL-SN-CCs, (**B**) FL-SN-CCs + DOs, (**C**) FL-NSN-CCs, (**D**) FL-NSN-CCs + DOs or in (**E**) control exosome-depleted α-MEM–Glutamax medium-only. (A–E) (**i**) Imaging flow cytometry analysis of EVs, with high gain mode acquisition and 60× magnification, plotting the distribution of particles positive or negative for the CD9 immunophenotype surface marker. (A–E) (**ii**) Representative fluorescent EVs images. Arrow, CD9-positive EV; Arrowhead, CD9-negative and SC-positive particle; BF, bright field; SC, size scatter. (A–E) (**iii**) Representative TEM images at 150 000× magnification. Arrow, EVs; Bar, EV diameter (nm). FL, feeder layer; SN, surrounded nucleolus; CCs, cumulus cells; NSN, not surrounding nucleolus; DO, denuded oocytes; EVs, extracellular vesicles; TEM, transmission electron microscopy.

Using imaging flow cytometry, we measured the release of EVs into the medium of FL-SN-CCs or FL-NSN-CCs after 15 h culture in the presence or absence of DOs (Fig. 1). As summarized in Table 1, there was no significant difference (*P* > 0.05) in the amount of EVs recorded across all four experimental conditions, which ranged from 460 000 to 710 000 particles/µl. The great majority (>96%) of EVs, regardless of the experimental condition, tested positive for the CD9 surface marker (Table 1; Fig. 1A–Di and A–Dii) and exhibited a diameter ranging from 18.5 to 54.9 nm upon TEM analysis (Table 1; Fig. 1A–Diii), indicating their exosome nature. Control exosome-depleted α-MEM–Glutamax medium-only samples showed the presence of an extremely low number of particles (236 ± 123 particles/µl), with just 4.5 ± 2.3 CD9-positive particles/µl (Fig. 1E).

**Table 1.** Characterization of extracellular vesicles released by feeder layers of mouse cumulus cells isolated from developmentally competent or incompetent oocytes in the presence or absence of denuded oocytes.

Experimental condition	Imaging flow cytometry			TEM		
	Particles/µl (mean ± SD)	CD9+ (%)	CD9− (%)	Diameter (nm)		
				Mean ± SD	Max	Min
FL-SN-CCs	713 208 ± 44 383	97.6	1.6	28.6 ± 5.9	45.7	18.5
FL-SN-CCs + DOs	592 845 ± 120 212	97.6	1.4	32.7 ± 5.8	42.4	22.3
FL-NSN-CCs	459 000 ± 301 949	96.3	1.4	32.2 ± 5.4	45.2	21.1
FL-NSN-CCs + DOs	501 871 ± 147 659	96.3	1.8	33.6 ± 5.4	54.9	23.3

Imaging flow cytometry quantification of extracellular vesicle (EV) concentration in the culture medium expressed as particles/µl; CD9+ or CD9− represent CD9-positive or -negative EVs, respectively; Transmission electron microscopy (TEM) sizing of EVs in nanometer (nm). Max, maximum value; Min, minimum value. FL-SN-CCs, feeder layer of cumulus cells isolated from developmentally competent oocytes; FL-SN-CCs + DOs, feeder layer of cumulus cells isolated from developmentally competent oocytes + denuded oocytes; FL-NSN-CCs, feeder layer of cumulus cells isolated from developmentally incompetent oocytes; FL-NSN-CCs + DOs, feeder layer of cumulus cells isolated from developmentally incompetent oocytes + denuded oocytes.

**Figure 2.**
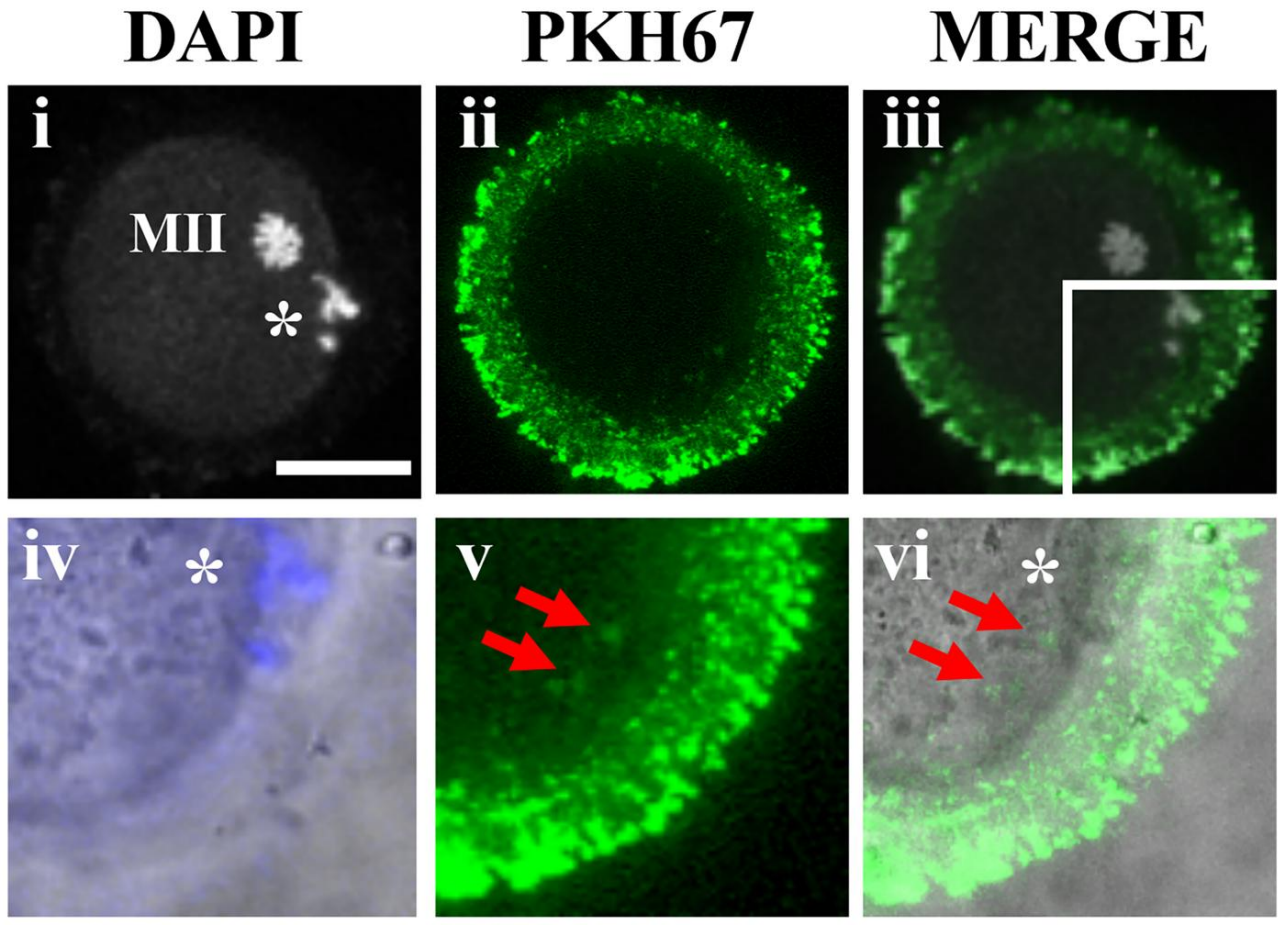
**Extracellular vesicles released by feeder layers of cumulus cells cross the mouse zona pellucida during the transition from germinal vesicle to metaphase II and some are internalized inside the ooplasm.** CC-free GV oocytes (DOs) were cultured for 15 h until they reached the MII phase in exosome-depleted α-MEM–Glutamax supplemented with PKH67-labelled EVs. (**i**–**iii**) Representative confocal image of an MII oocyte showing the presence of numerous EVs inside the ZP layer; enlargement of the same MII oocyte, overlapping the bright-field image with DAPI (**iv**), showing the presence of PKH67-labelled EVs crossing a region of the ZP and also inside the ooplasm (**v**, **vi**; red arrows). The complete z-stack images are shown in Supplementary Video S1. Bar, 50 µm; MII, metaphase II; *, polar body I. Control samples in which oocytes were matured in exosome-depleted α-MEM-Glutamax without EVs and without PKH67, in α-MEM without EVs but with PKH67 or in α-MEM supplemented with PKH67-unlabelled EVs are shown in Supplementary Fig. S1.

As oocytes are surrounded by a ZP, we investigated whether the EVs could pass through this glycoprotein layer. Therefore, DOs were cultured to MII for 15 h in 300 µl α-MEM containing PKH67-labelled EVs. Confocal microscope analysis revealed the presence of several fluorescent vesicles within the ZP (Fig. 2ii and iii), some of which were also visible inside the ooplasm (Fig. 2v and vi; Supplementary Video S1). Control samples in which DOs were matured in exosome-depleted α-MEM–Glutamax medium without EVs and without PKH67 (Supplementary Fig. S1i and ii), without EVs but with PKH67 (Supplementary Fig. S1iii and iv), or in α-MEM supplemented with PKH67-unlabelled EVs (Supplementary Fig. S1v and vi), did not exhibit the presence of fluorescent particles.

### Analysis of EVs miRNA content

**Figure 3.**
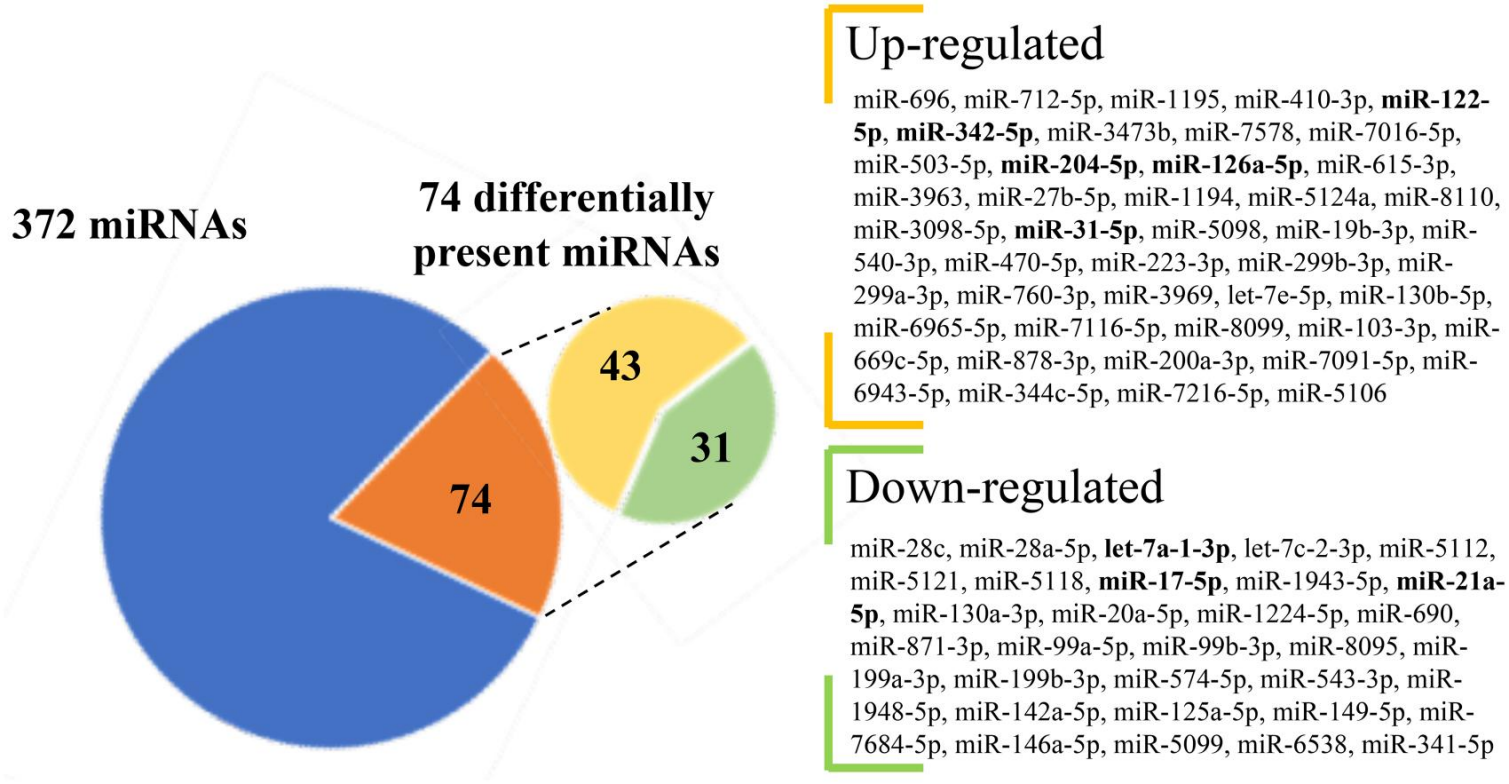
**miRNAs differentially present in the comparison between extracellular vesicles collected from the medium of feeder layers of mouse cumulus cells isolated from developmentally competent or incompetent oocytes in the presence denuded oocytes.** Up- and downregulated miRNAs are listed according to their fold-change. Bold font, 5 up- and 3 down-regulated miRNAs involved in ovarian functions. miRNA: microRNA.

Our next aim was to identify the miRNA cargo of the EVs. Therefore, we compared the miRNome of EVs released by FL-SN-CCs + DOs with that of EVs released by FL-NSN-CCs + DOs. RNA sequencing by NGS revealed a total of 372 miRNAs, 74 of which were significantly differentially expressed. Of these, 43 were upregulated and 31 were downregulated in FL-SN-CCs + DOs (Fig. 3; Supplementary Table S2). The expression of three of the most downregulated (i.e. miR-28c, let-7a-1-3p, miR-17-5p) and two of the most upregulated (i.e. miR-342-5p, miR-696) miRNAs were validated by qPCR (Supplementary Fig. S2).

### Identification of miRNA target genes and pathways

A PubMed search of the role of these 74 differentially expressed miRNAs showed that most are circulating. Among these, eight miRNAs (five up- and three down-regulated; Fig. 3) have previously been associated with ovarian functions: miR-126a-5p (GCs apoptosis) (Li *et al.*, 2019); miR-204-5p (GCs apoptosis, oocyte developmental competence) (Pasquariello *et al.*, 2020); let-7a-1-3p (GCs and CCs apoptosis) (Salilew-Wondim *et al.*, 2014; Zhang *et al.*, 2019a); miR-342-5p (GV-to-MII transition) (Xiong *et al.*, 2022); miR-21a-5p (GCs and CCs apoptosis inhibition, cumulus expansion, oocyte developmental competence) (Pan and Li, 2018); miR-31-5p (GCs proliferation, cumulus expansion, steroid synthesis) (Zhang *et al.*, 2019b); miR-17-5p (GCs proliferation, corpus luteum differentiation) (Zhang *et al.*, 2019a); and miR-122-5p (LHR expression) (Menon *et al.*, 2017). To decipher with more precision the regulatory role that these eight miRNAs might have during the GV-to-MII transition and in the acquisition of oocyte developmental competence, they were further analyzed by using DIANA mirPath. This analysis identified 648 predicted or experimentally validated target genes (Supplementary Table S3).

Then, a systematic bibliographical search yielded 935 publications, in which 71 genes were found to be involved in meiosis resumption (N = 24 genes), cumulus expansion (N = 23), fertilization (N = 1) and oocyte developmental competence (N = 23). Table 2 shows the target genes and the corresponding bibliographical references for each miRNA. miR-342-5p was not associated with target genes specific to ovarian function and thus was not further analyzed.

**Table 2.** List of 71 microRNA target genes playing a role in meiosis resumption, cumulus expansion, fertilization, or oocyte developmental competence in mammals.

miRNA	Meiosis resumption	Cumulus expansion	Fertilization	Oocyte developmental competence
miR-17-5p	*Actb* ^1^, *Apc*^2,3^*, Cdk4*^4^*, Fyn*^5^*, Gbf1*^6^*, Hif1a*^7^*, Limk1*^8^*, Pak1*^9^*, Pak2*^10^*, Pde3a*^11^	*Ar* ^25^ *, Ccnd2* ^26^ *, Col1a1* ^27^ *, Creb1* ^28^ *, Cxcl12* ^29^ *, Bmp4* ^30^ *, Bmpr2* ^31^ *, E2f1* ^32^ *, Foxo1* ^33^ *, Gja1* ^34^ *, Ldlr* ^35^ *, Mdm2* ^36^ *, Runx1* ^37^ *, Smad4* ^38^ *, Tgfbr1* ^39^	*Plcb1* ^47^	*Akt1* ^48^ *, Cdk2* ^49^ *, Ccne1* ^50^ *, Irf1* ^51^ *, Klf4* ^52^ *, Kras* ^53^ *, Lats1* ^54^ *, Pard6b* ^55^ *, Pfkp* ^56^ *, Pikfyve* ^57^ *, Setd2* ^58^ *, Sos1* ^48^ *, Spp1* ^48^ *, Stat3* ^59^
miR-21a-5p		*Yap1* ^40^		
let-7a-1-3p	*Apc* ^2,3^ *, Cdc42* ^12^ *, RhoA* ^13^	*Foxo1* ^33^ *, Smad4* ^38^		*Cul1* ^60^ *, Kdm6a* ^61^
miR-31-5p	*Cdc42* ^12^ *, Hif1a* ^7^ *, Hdac2* ^14^ *, Pak2* ^10^ *, Pak4* ^15^ *, Ptk2b* ^16^ *, Ralb* ^17^ *, Sirt1* ^18^	*Cxcl12* ^29^ *, Smad4* ^38^		*Sos1* ^48^ *, Stat3* ^59^
miR-122-5p	*Apc* ^2,3^ *, Bub1b* ^19^ *, Cdc14b* ^3,20^ *, Fzr1* ^2^	*Bmpr2* ^31^ *, Ctgf* ^41^ *, E2f1* ^32^ *, Foxo1* ^33^ *, Smad4* ^38^ *, Tgfbr1* ^39^		*Myc* ^62^ *, Setd2* ^58^
miR-126-5p	*Pak2* ^10^	*Erbb4* ^42^	*Plcb1* ^47^	*Cry1* ^63^ *, Sos1* ^48^
miR-204-5p	*Apc* ^2,3^ *, Arf6* ^21^ *, Npr2* ^22^ *, Pde3a* ^11^ *, Rab8a* ^23^ *, Rab14* ^24^ *, Sirt1* ^18^	*Cnot6l* ^43^ *, Creb1* ^28^ *, Dnm2* ^44^ *, Gja1* ^34^ *, Mdm2* ^36^ *, Mmp2* ^45^, *Runx2*^46^		*Adipor2* ^64^, *Camk1d*^65^*, Cnot7*^66^*, Esr1*^67^*, Kras*^53^*, Setd1b*^68^*, Setd2*^58^*, Sos1*^48^
Total no. of genes	24	23	1	23

miRNA, microRNA.

(1) Wei *et al.*, 2018; (2) Holt *et al.*, 2012; (3) Bai *et al.*, 2020; (4) Dong *et al.*, 2021; (5) Luo *et al.*, 2010; (6) Zou *et al.*, 2021a; (7) Li *et al.*, 2020a; (8) Duan *et al.*, 2018a; (9) Lin *et al.*, 2010; (10) Zeng *et al.*, 2023; (11) Strączyńska *et al.*, 2022; (12) Mei *et al.*, 2022; (13) Zhang *et al.*, 2014; (14) Ma and Schultz, 2016; (15) He *et al.*, 2019; (16) Meng *et al.*, 2018; (17) Sun *et al.*, 2021; (18) Ferreira *et al.*, 2022; (19) Wei *et al.*, 2010; (20) Schindler and Schultz, 2009; (21) Duan *et al.*, 2018b; (22) Arroyo *et al.*, 2020; (23) Pan *et al.*, 2019; (24) Zou *et al.*, 2021b; (25) Walters *et al.*, 2012; (26) Dai *et al.*, 2021; (27) Shen *et al.*, 2022; (28) Sirotkin *et al.*, 2019; (29) Zhang *et al.*, 2018; (30) Tian *et al.*, 2022; (31) Liu *et al.*, 2018; (32) Morrell *et al.*, 2019; (33) Li *et al.*, 2021; (34) Ackert *et al.*, 2001; (35) Meng *et al.*, 2022; (36) Haraguchi *et al.*, 2019; (37) Liu *et al.*, 2010; (38) Li *et al.*, 2020b; (39) Wang *et al.*, 2021; (40) Sun and Diaz, 2019; (41) Chermuła *et al.*, 2020; (42) Veikkolainen *et al.*, 2020; (43) Dai *et al.*, 2021; (44) Mihalas *et al.*, 2020; (45) Luddi *et al.*, 2018; (46) Lee-Thacker *et al.*, 2018; (47) Igarashi *et al.*, 2007; (48) Artini *et al.*, 2017; (49) Wang and Kim, 2016; (50) Krivega *et al.*, 2015; (51) Kim *et al.*, 2011; (52) Wang *et al.*, 2016; (53) Fan *et al.*, 2008; (54) Lorthongpanich *et al.*, 2013; (55) Alarcon, 2010; (57) Ikonomov *et al.*, 2011; (58) Li *et al.*, 2020c; (59) Ghanem *et al.*, 2020; (60) Benesova *et al.*, 2016; (61) Bai *et al.*, 2018; (62) Asami *et al.*, 2023; (63) Amano *et al.*, 2009; (64) Richards *et al.*, 2012; (65) Scarica *et al.*, 2019; (66) Ma *et al.*, 2015; (67) Chermuła *et al.*, 2019; (68) Bilmez *et al.*, 2022.

**Figure 4.**
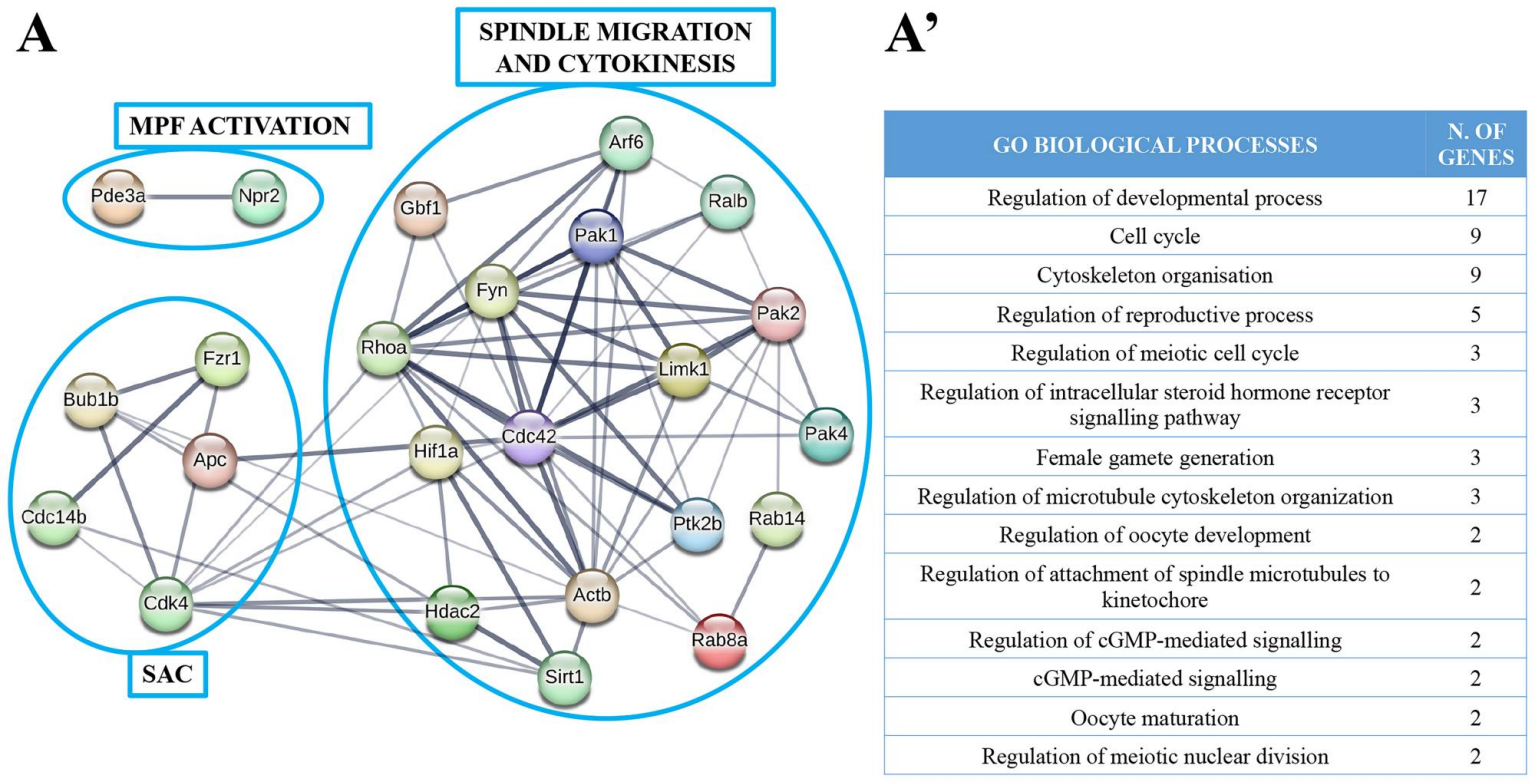
**Target genes of mouse cumulus cell-derived microRNAs during meiosis resumption and the biological processes involved.** MicroRNA-target genes involved in meiosis resumption (**A**), with the corresponding biological processes identified by STRING (**A′**). MPF: maturation promoting factor, SAC: spindle assembly checkpoint.

Figure  4A shows that the 24 target genes involved in meiosis resumption are specifically associated with the spindle assembly checkpoint (Schindler and Schultz, 2009; Wei *et al.*, 2010; Holt *et al.*, 2012; Bai *et al.*, 2020; Dong *et al.*, 2021), spindle migration and cytokinesis during oocyte maturation (Lin *et al.*, 2010; Luo *et al.*, 2010; Zhang *et al.*, 2014; Meng *et al.*, 2018; Duan *et al.*, 2018a,b; Wei *et al.*, 2018; He *et al.*, 2019; Pan *et al.*, 2019; Li *et al.*, 2020a; Sun *et al.*, 2021; Zou *et al.*, 2021a,b; Ferreira *et al.*, 2022; Mei *et al.*, 2022; Zeng *et al.*, 2023) and meiosis resumption through MPF (maturation promoting factor) activation (Arroyo *et al.*, 2020; Strączyńska *et al.*, 2022). These results were supported by a further analysis with STRING that ascribed the genes to 14 main biological processes (Fig. 4A′ and Supplementary Table S4) involved in the regulation of cytoskeleton organization, attachment of spindle microtubules to the kinetochore, meiotic cell cycle, intracellular steroid hormone receptor signalling pathway, female gamete generation, oocyte development and maturation, cGMP-mediated signalling, and meiotic nuclear division.

**Figure 5.**
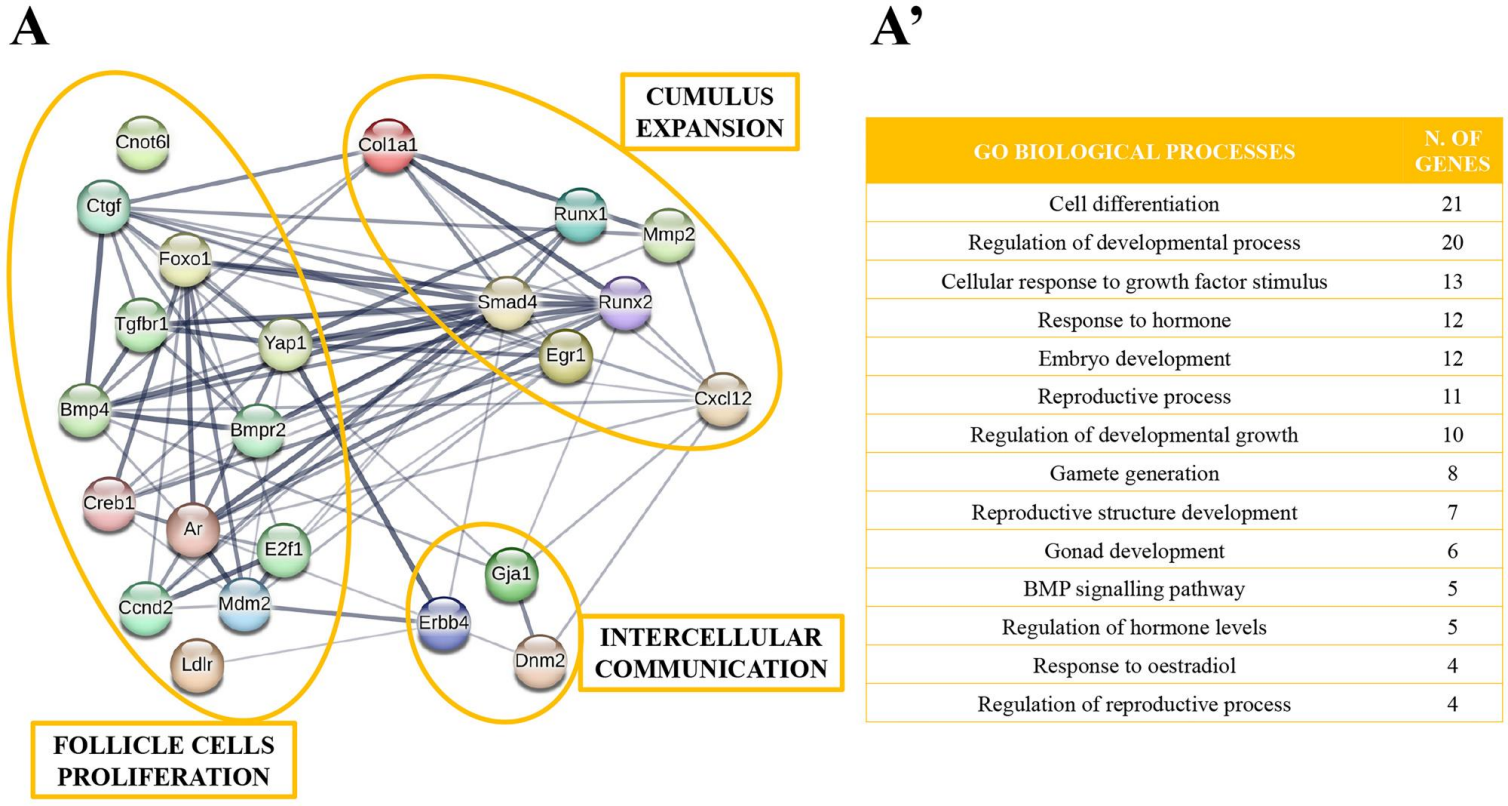
**Target genes of mouse cumulus cell-derived microRNAs involved in follicle cell proliferation and cumulus expansion and the biological processes involved.** MicroRNA-target genes involved in follicle cells proliferation and cumulus expansion (**A**), with the corresponding biological processes identified by STRING (**A′**). BMP: bone morphogenetic protein.

The 23 target genes of the group ‘follicle cells proliferation and expansion’ (Fig. 5A) were, more specifically, ascribed to follicle cells proliferation (Walters *et al.*, 2012; Han *et al.*, 2013; Liu *et al.*, 2018; Haraguchi *et al.*, 2019; Morrell *et al.*, 2019; Sirotkin *et al.*, 2019; Sun and Diaz, 2019; Chermuła *et al.*, 2020; Dai *et al.*, 2021; Li *et al.*, 2021; Wang *et al.*, 2021; Meng *et al.*, 2022; Tian *et al.*, 2022), cumulus expansion (Liu *et al.*, 2010; Siddappa *et al.*, 2015; Lee-Thacker *et al.*, 2018; Luddi *et al.*, 2018; Zhang *et al.*, 2018; Li *et al.*, 2020b; Shen *et al.*, 2022), and intercellular communication (Ackert *et al.*, 2001; Mihalas *et al.*, 2020; Veikkolainen *et al.*, 2020). When analyzed with STRING, these genes were associated with biological processes such as cell differentiation and response to growth factor stimuli, embryo development, reproductive and developmental processes, gonad development and gamete generation, and regulation of hormone levels (Fig. 5A′ and Supplementary Table S4).

*Plcb1* is the only miRNA target gene with a putative role in fertilization (Table 2), when, by acting in combination with a sperm-derived phospholipase C zeta, induces oocyte activation (Igarashi *et al.*, 2007).

**Figure 6.**
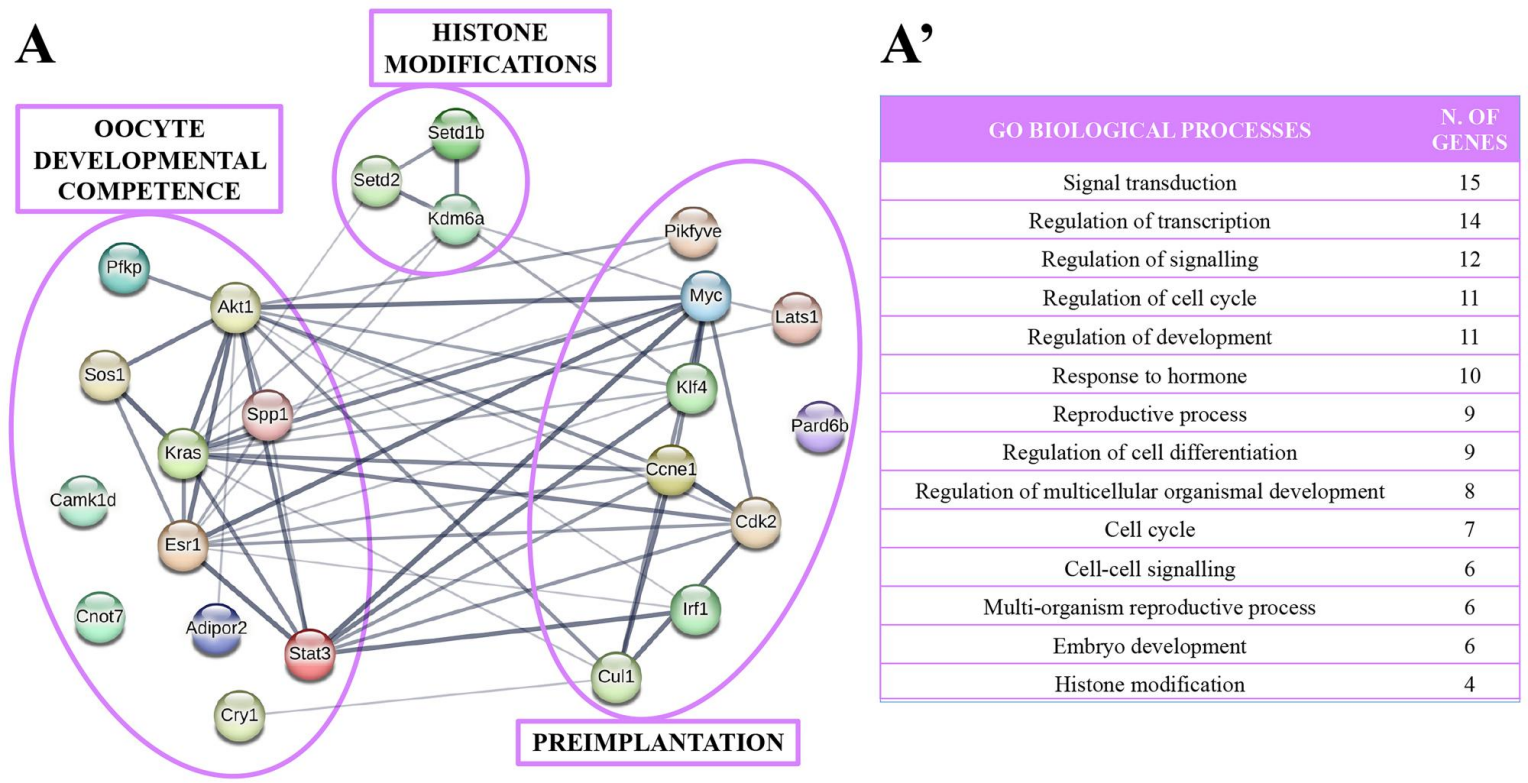
**Target genes of mouse cumulus cell-derived microRNAs involved in oocyte developmental competence and the biological processes involved.** MicroRNA-target genes involved in oocyte developmental competence (**A**), with the corresponding biological processes identified by STRING (**A′**).

The 23 genes belonging to the ‘oocyte developmental competence’ group (Fig. 6A) were attributed to oocyte developmental competence (Fan *et al.*, 2008; Amano *et al.*, 2009; Richards *et al.*, 2012; Ma *et al.*, 2015; Artini *et al.*, 2017; Chermuła *et al.*, 2019; Scarica *et al.*, 2019; Ghanem *et al.*, 2020; Shen *et al.*, 2020), histone modifications (Bai *et al.*, 2018; Li *et al.*, 2020c; Bilmez *et al.*, 2022) and preimplantation (Riego *et al.*, 1995; Alarcon, 2010; Ikonomov *et al.*, 2011; Kim *et al.*, 2011; Lorthongpanich *et al.*, 2013; Krivega *et al.*, 2015; Benesova *et al.*, 2016; Wang *et al.*, 2016; Wang and Kim, 2016; Asami *et al.*, 2023). STRING analysis related these genes to biological processes such as signal transduction, regulation of transcription and cell cycle, cell–cell signalling, reproductive process, histone modification and embryo development (Fig. 6A′ and Supplementary Table S4).

## Discussion

Our results show that during the *in vitro* GV-to-MII transition in mice, both FL-SN-CCs and FL-NSN-CCs release a large amount of EVs into the medium. After 15 h culture, these EVs are observed to cross the ZP or are already present inside the ooplasm. The permeability of the ZP to cell-derived structures, such as EVs, is not a novelty. The ZP has been shown to be permeable to a variety of molecules, depending on both their size and chemical properties. For instance, small viruses (Mateusen *et al.*, 2007; Romeo *et al.*, 2020), ferritin (Hastings *et al.*, 1972), and immunoglobulins (Sellens and Jenkinson, 1975) of 12–20 nm in size may cross this glycoprotein layer. More recently, four fluorescence microscopy studies have analyzed the passage of PKH67-labeled EVs through the ZP. Three studies have shown that bovine follicular fluid-derived EVs of <200 nm in size, added to the medium during COC maturation or preimplantation development, cross the ZP of both blastocysts (da Silveira *et al.*, 2017; Pavani *et al.*, 2018) or oocytes and reach the ooplasm (Uzbekova *et al.*, 2020). In another study, EVs were added to the culture medium of two-cell nuclear transfer porcine embryos, and they passed through the ZP and entered single blastomeres (Saadeldin *et al.*, 2014). In addition, fluorescent inert microspheres with a size <200 nm passed through the ZP of porcine preimplantation embryos, whereas those >200 nm remained outside (Mateusen *et al.*, 2004). Altogether, these studies suggest that ZP permeability is limited to particles smaller than 200 nm in diameter. This size range corresponds to the majority of EVs released by FL-CCs in our experimental conditions, which are within the size range of exosomes (Doyle and Wang, 2019). In our study, after crossing through the ZP, we detected EVs in the MII cytoplasm, suggesting endocytosis as a delivery mechanism. However, we cannot exclude the possibility of a direct release of EV content after fusion with the oolemma. Further evidence of EV uptake has been found in culture systems where the medium was supplemented with small EVs isolated from the follicular fluid. This resulted in improved oocyte maturation and preimplantation development (da Silveira *et al.*, 2015, 2018).

**Figure 7.**
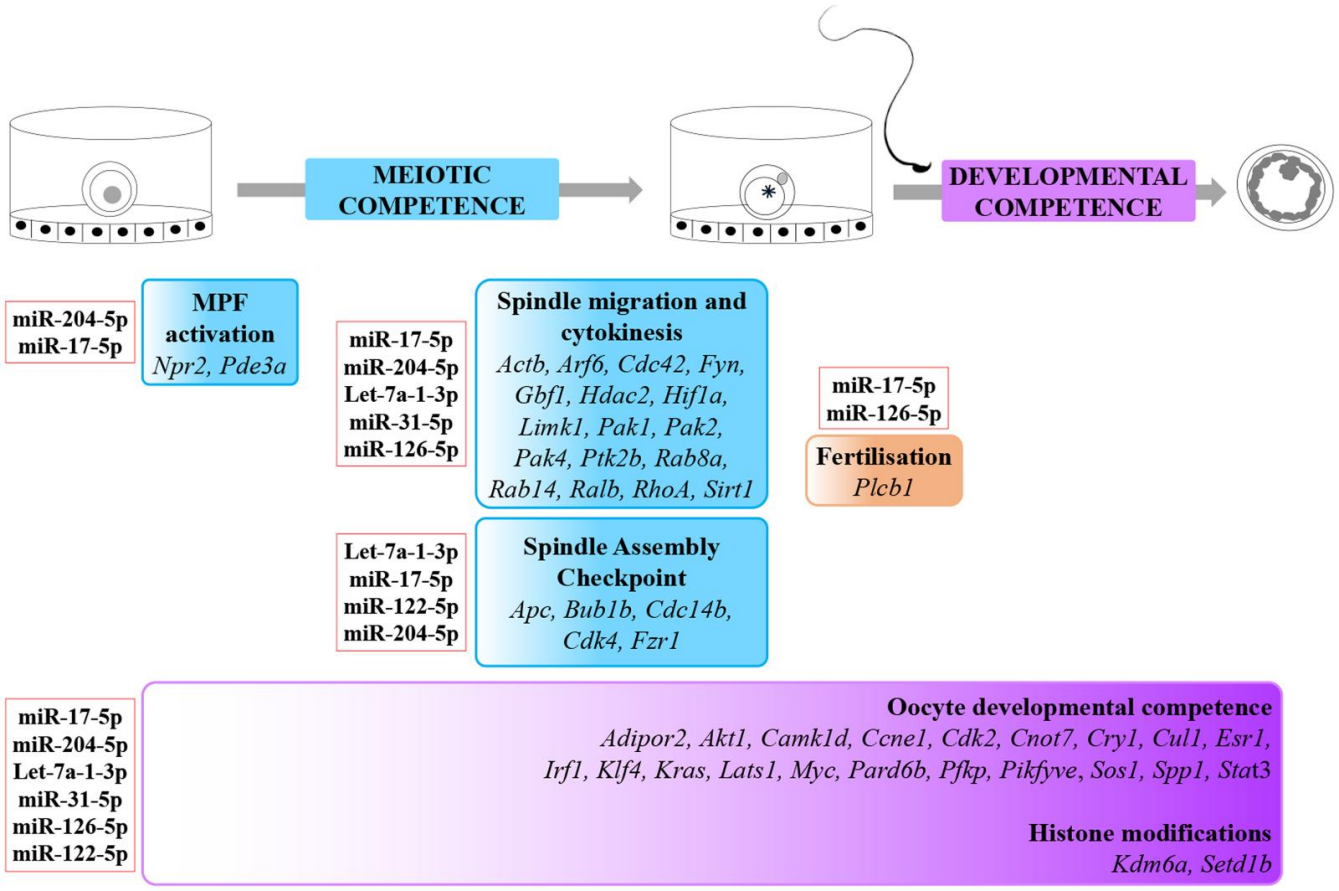
**A proposed scenario summarizing the role of candidate cumulus cell-derived microRNAs and the genes that they regulate during the acquisition of oocyte competence.** Red frame box: candidate cumulus cell-derived miRNAs. Genes the mRNAs regulate during oocyte meiotic resumption (MPF activation, spindle assembly checkpoint, migration, and cytokinesis; blue boxes), fertilization (orange box), and acquisition of oocyte developmental competence (purple box) are shown. miRNA, microRNA; MPF, maturation promoting factor.

Our study focuses on identifying factors within EVs that contribute to oocyte developmental competence. The primary focus is on their miRNA content in view of their role as pleiotropic and ubiquitous post-translational regulators of gene expression (Brennecke *et al.*, 2005). We found seven miRNAs (i.e. miR-17-5p, miR-21a-5p, let-7a-1-3p, miR-31-5p, miR-122-5p, miR-126-5p, and miR-204-5p) that regulate 71 target genes involved in folliculogenesis and the acquisition of oocyte meiotic and developmental competence (Fig. 7). Of these, 24 target genes are involved in key events necessary for oocyte meiotic resumption (i.e. MPF activation, spindle assembly checkpoint, migration, and cytokinesis) (Fig. 7, blue boxes), 1 gene for fertilization (Fig. 7, orange box), and 23 genes are involved in the acquisition of oocyte developmental competence (Fig. 7, purple box).

At this stage, we acknowledge that our work was limited to a small sub-group of 8 out of 74 identified miRNAs. These were chosen because they had already been experimentally related to ovarian functions. We suggest that the remaining 66 miRNAs (Fig. 3 and Supplementary Table S2) could be valuable for investigating their role in regulating target genes of interest. Additionally, they could aid in interpretating data resulting from other experimental settings. As an example, we anticipate that this list of miRNAs has proven useful beyond our SN/NSN model study of oocyte developmental competence/incompetence. Recently, we discovered that miR-28c, the most down-regulated miRNA on our list, is also linked to oocyte developmental competence in a different study model: the ZP3-Cre Cabin1-knockout mouse (KO), which experiences preimplantation development arrest at the 2- to 4-cell stage (Smith *et al.*, 2022). Similar to the SN/NSN model, miR-28c was significantly down-regulated in EVs released by FL-CCs of wild-type compared to FL-CCs of developmentally incompetent KO oocytes (our unpublished data). Although further analysis with Diana Tools did not identify any target genes with a regulative role in the ovary, miR-28c is an interesting marker of oocyte developmental competence and worth further study.

In summary, our study shows that FL-CCs release EVs during the *in vitro* GV-to-MII transition in mice. The majority of these EVs have a diameter of less than 200 nm, indicating their potential to cross the ZP, which is consistent with their detection inside the ooplasm by confocal microscopy. These EVs contain a wide range of miRNAs that are differentially expressed in FL-SN-CCs versus FL-NSN-CCs, seven of which are potentially capable of modulating the expression of selected genes that contribute to oocyte developmental competence. Identifying this group of miRNA target genes will enable future studies to quantify their expression in oocytes matured on either FL-SN-CCs or FL-NSN-CCs or in their derived conditioned media. In conclusion, our results have identified a group of miRNAs that may act as regulatory factors, and whose role is now to be experimentally verified by selective inactivation.

## Supplementary Material

gaae019_Supplementary_Data

## Data Availability

Data underlying this article are available in the article, in its online supplementary material and in FigShare, at DOI: 10.6084/m9.figshare.25324048.

## Author Biography

**Dr. Giulia Fiorentino:** Dr. Fiorentino holds a Master’s degree in molecular biology and genetics (2017) and a PhD in bioengineering (2021). In 2022, aged 29 years, she won a competition for a 3-year junior researcher position within the National Operative Program for Research and Innovation at the University of Pavia (Italy). This marked the beginning of her independent career. Her research focuses on elucidating the cytological and molecular factors involved in the acquisition of mammalian oocytes’ developmental competence. This is achieved either by *in vitro* culture systems or, using confocal and tomographic imaging, through the reconstruction of 3D *in silico* models of the ovary.
