# Supplementary material for: Extracellular vesicles secreted by cumulus cells contain microRNAs that are potential regulatory factors of mouse oocyte developmental competence
Source: Mol Hum Reprod. 2024 May 14;30(6):gaae019. doi: 10.1093/molehr/gaae019 (PMC12054941; doi:10.1093/molehr/gaae019)
Supplement: gaae019_Supplementary_Data [file gaae019_supplementary_data.zip › Supplementary_Information_.gaae019.pdf]

Supplementary information

Extracellular vesicles secreted by cumulus cells contain microRNAs that are potential regulatory factors of mouse oocyte developmental competence

Giulia Fiorentino[1,†], Valeria Merico[1,†], Mario Zanoni[1], Sergio Comincini[2], Daisy Sproviero[3], Maria Garofalo[4], Stella Gagliardi[4], Cristina Cereda[5], Chih-Jen Lin[6], Federica Innocenti[7], Marilena Taggi[7], Alberto Vaiarelli[7], Filippo Maria Ubaldi[7], Laura Rienzi[7,8], Danilo Cimadomo[7], Silvia Garagna[1], Maurizio Zuccotti[1]

[†]: These authors contributed equally to this work.

Supplementary Figures and Tables:

- **Supplementary Fig. S1.** Confocal microscope analysis of representative metaphase II oocytes cultured for 15 hr in exosome-depleted α-MEM-Glutamax medium without extracellular vesicles and without PKH67 (i-ii), α-MEM without extracellular vesicles but with PKH67 (iii-iv) or in α-MEM supplemented with PKH67-unlabelled extracellular vesicles (v-iv).
- **Supplementary Fig. S2.** Fold-change expression of three down- (green: miR-28c, let-7a-1-3p, miR-17-5p) and two up- (red: miR-342-5p, miR-696) regulated microRNAs.
- **Supplementary Table S1.** miRCURY LNA miRNA PCR Assays primers used for qPCR validation. hsa-miR-16-5p was used as internal control for normalisation.

The following Supplementary Data Files are available as separate files:

- **Supplementary Table S2**. Differentially expressed microRNAs in the comparison between extracellular vesicles collected from the medium of feeder layers of mouse cumulus cells isolated from developmentally competent or incompetent oocytes in the presence denuded oocytes. – format excel
- **Supplementary Table S3**. Predicted or experimentally validated target genes – format excel
- **Supplementary Table S4**. Association of genes and biological processes obtained with STRING analysis. Lines highlighted in green represent the most relevant biological processes associated with meiosis resumption – format excel
- **Supplementary Video S1.** 175 Z-stack confocal images (z-step 0.5 µm) of the metaphase II oocyte shown in Figure 3 – format mp4
- Next-generation sequencing data are available in FigShare, at DOI:

  https://doi.org/10.6084/m9.figshare.25324048.v1

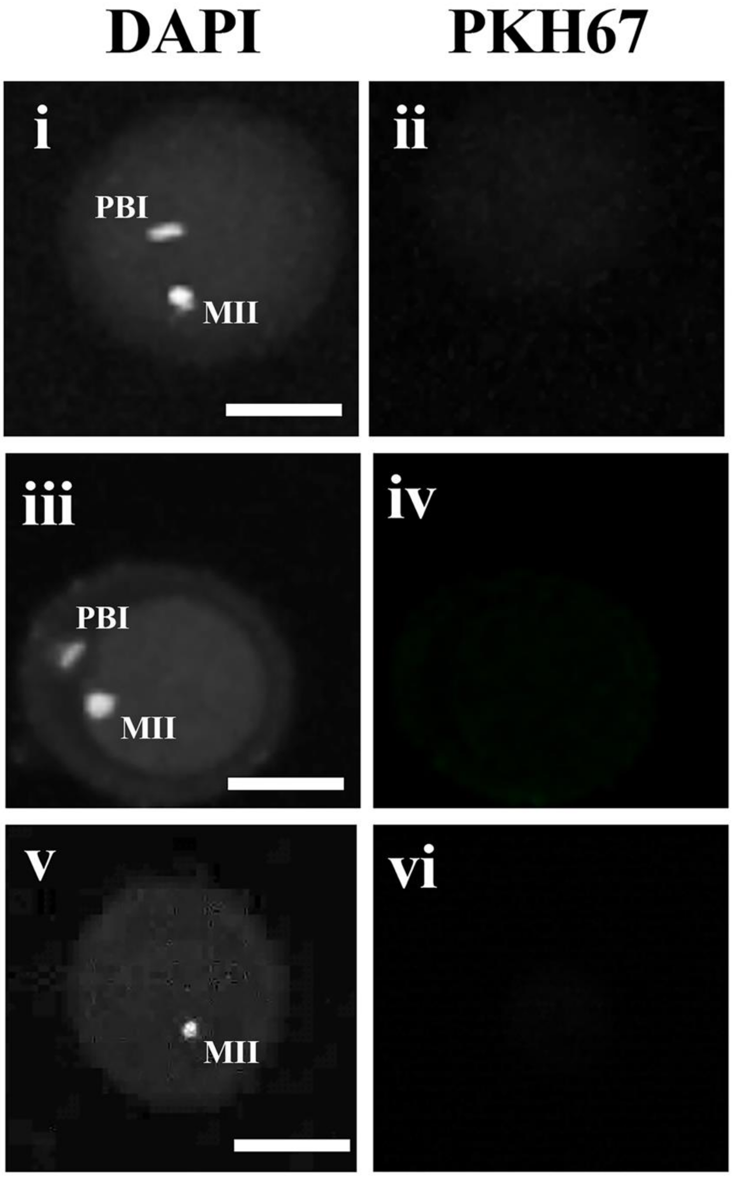

Supplementary Fig. S1. Confocal microscope analysis of representative metaphase II oocytes cultured for 15 hr in exosome-depleted α-MEM-Glutamax medium without extracellular vesicles and without PKH67 (i-ii), α-MEM without extracellular vesicles but with PKH67 (iii-iv) or in α-MEM supplemented with PKH67-unlabelled extracellular vesicles (v-iv). MII, Metaphase II; PBI, Polar Body I; Bar, 50 µm.

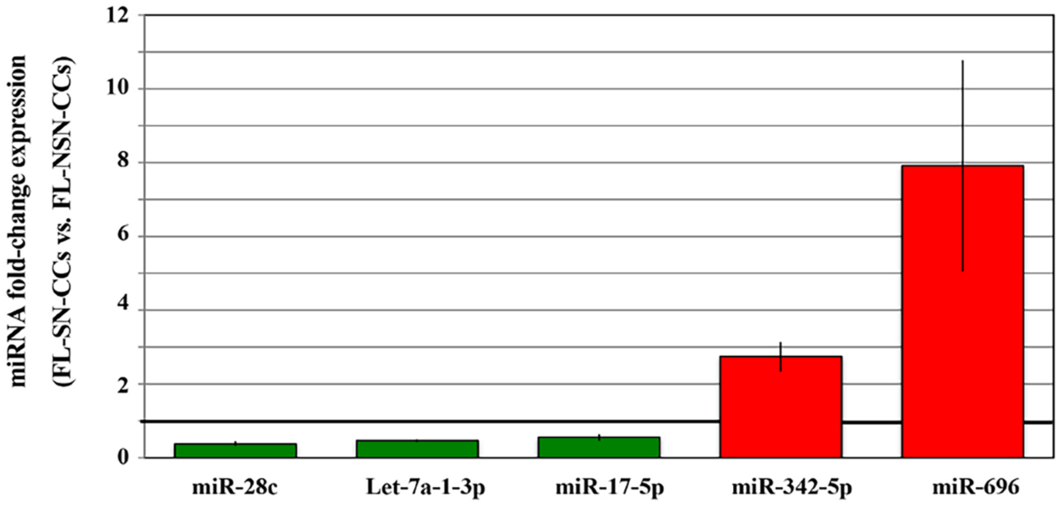

Supplementary Fig. S2. Fold-change expression of three down- (green: miR-28c, let-7a-1-3p, miR-17-5p) and two up- (red: miR-342-5p, miR-696) regulated microRNAs. Internal control hsa-miR-16-5p was set at 1.

Supplementary Table S1. miRCURY LNA miRNA PCR Assays primers used for qPCR validation. hsa-miR-16-5p was used as internal control for normalisation.

miRCURY LNA miRNA PCR Assay	Cat N.
mmu-miR-28c	YP02110936
mmu-miR-696	YP02102080
mmu-let-7a-1-3p	YP00205586
mmu-miR-17-5p	YP02119304
mmu-miR-342-5p	YP00205185
hsa-miR-16-5p	YP00205702

Supplementary Video S1. 175 Z-stack confocal images (z-step 0.5 µm) of the metaphase II oocyte shown in Figure 3. Bright-field, blue (DAPI-stained chromosomes) and green (PKH67-labelled extracellular vesicles) channels are overlapped to generate the sequence images. Red arrows, internalised extracellular vesicles.

Next-generation sequencing data underlying this article are available in FigShare, at DOI: 10.6084/m9.figshare.25324048
